# Maxillary sinus aeration analysis using computational fluid dynamics

**DOI:** 10.1038/s41598-022-14342-3

**Published:** 2022-06-20

**Authors:** Dmitry Tretiakow, Krzysztof Tesch, Karolina Markiet, Andrzej Skorek

**Affiliations:** 1 Department of Otolaryngology, Medical University of Gdansk, Gdansk, Poland; 2 Faculty of Mechanical Engineering and Ship Technology, Gdansk University of Technology, Gdansk, Poland; 3 II Department of Radiology, Medical University of Gdansk, Gdansk, Poland

**Keywords:** Computational biology and bioinformatics, Anatomy, Medical research, Pathogenesis, Risk factors, Diseases, Respiratory tract diseases, Signs and symptoms, Respiratory signs and symptoms

## Abstract

The maxillary sinus aeration using the computational fluid dynamics (CFD) method based on individual adult patients’ computed tomography (CT) scans were analyzed. The analysis was based on CT images of 4 patients: one with normal nose anatomy and three with nasal septal deviation (NSD) and concha bullosa (CB). The CFD simulation was performed using the Reynolds-Average Simulation approach and turbulence closure based on linear eddy viscosity supplemented with the two-equation *k*-$\omega$ SST model. As a result, it was found that the lower part of NSD has the most significant impact on the airflow change within the maxillary sinuses compared to CB and the upper part of NSD. In a healthy nose, the airflow in the sinuses is continuous, while NSD and CB change this flow into pulsatile. Multiple changes in the direction of flow during one respiratory phase were observed. The flow intensity within the maxillary sinus opening is lower on the NSD side. The concept of vorticity measure is introduced to evaluate and compare various patients qualitatively. Typically, the lowest values of such measures are obtained for healthy airways and the highest for pathological changes in the nasal cavity.

## Introduction

Due to its frequency of occurrence, deterioration in patients’ quality of life, coexistence/induction of lower respiratory tract diseases, and the enormous costs associated with its therapy, chronic sinusitis is becoming a social dimension. Despite the development of microbiological, histological, and radiological diagnostics, the cause of sinusitis remains difficult to define. The combination of topical medications and surgical procedures remains the gold standard of therapy^1–3^. The development of micro-invasive endoscopic techniques has led to a change in the therapeutic approach to nasal diseases. Modern techniques (Endoscopic Sinus Surgery, ESS), Functional Endoscopic Sinus Surgery (FESS) aim to clear the nose and restore (re-create) the physiological sinonasal connection^4–6^. The main challenge and, at the same time, the difficulty of these procedures is the desire to minimize tissue injuries while at the same time individualizing the scope of the operation^7–9^. Preoperative, up-to-date radiological evaluation of the craniofacial cavity is used to accurately assess the surgical field and determine potentially hazardous sites^10,11^. The main surgical procedures focus on the ostiomeatal complex, the physiological pathway to the anterior group of paranasal sinuses (anterior ethmoid, maxillary and frontal sinuses). The defense mechanisms against the development of inflammatory processes in this group of sinuses are conditioned by the presence of barriers, e.g., anatomical (complex structure of the mouth-ductal complex), histological (mucociliary transport), cellular, chemical (pH change, oxygen content change), and molecular^12–14^. The problem of airflow through the nasal cavity, the nose’s lateral wall, and the sinuses’ inside are entirely ignored in clinical practice. It is known that both pathogenic microbes and environmental pollutants enter the nose with air, but the mechanism by which air flows through the nose and sinuses is not known. It is known that mucociliary transport is directed towards the ostium of the sinus and the nasopharynx. At the same time, the role of the direction of air movement remains unclear: does it interact with this mechanism, or is it opposed to it? Finally, the influence of the pathology of the nasal cavity inside (e.g., nasal septum deviation (NSD), nasal turbinate hypertrophy, presence of the concha bullosa) and the consequences of surgical interventions (e.g., sinus widening) on the flow of air through the nose are still unclear^15–18^.

This study presents the results of the evaluation aeration activity in the human maxillary sinus using the CFD simulation method in a normal nasal cavity and in three with some kind of pathology. Moreover, the vorticity measures, which show clear correlations between patients in the norm and pathological situations quantitatively were adopted.

## Results

### Patients and scanning characteristics

**Figure 1:**
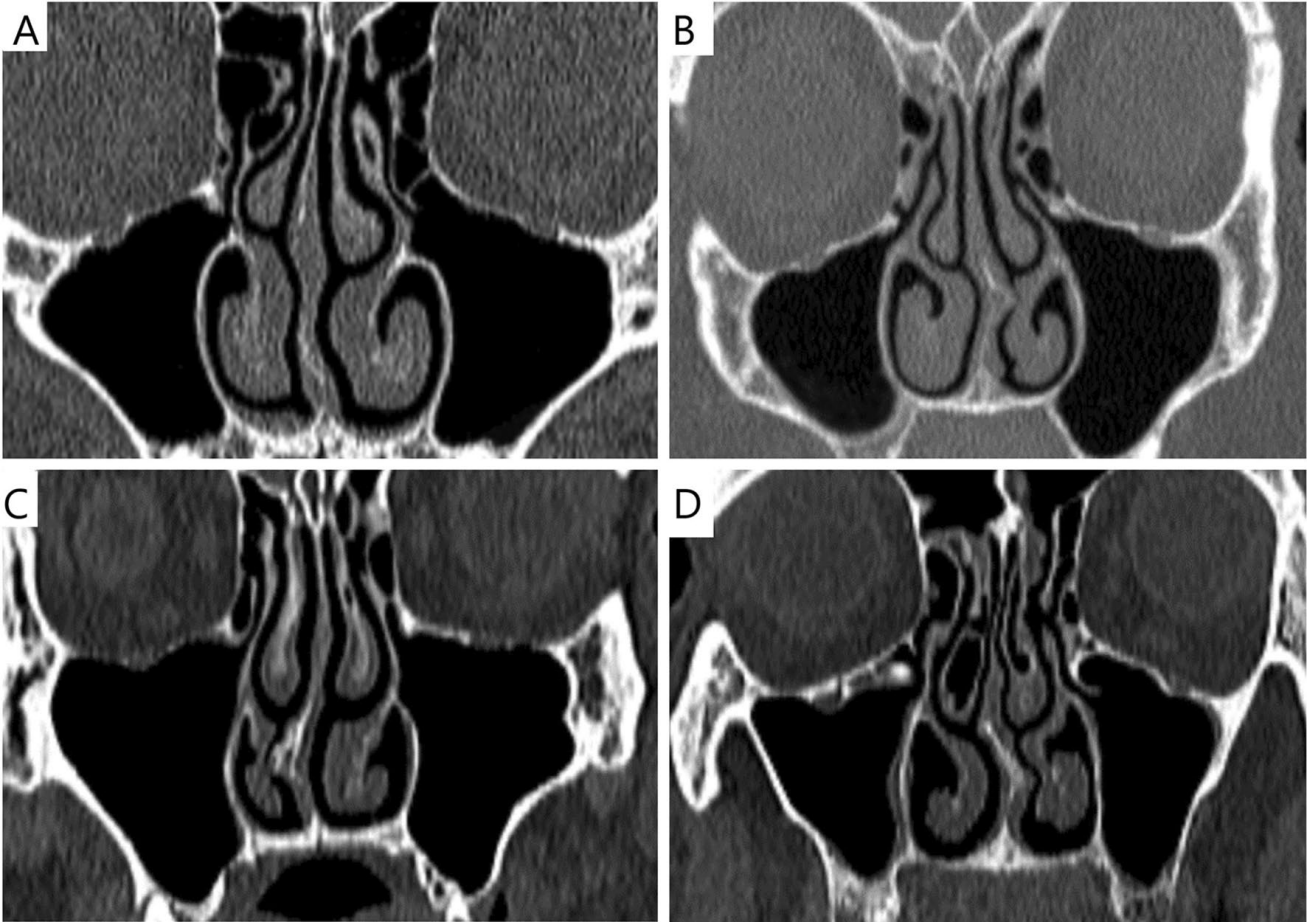
CT scans, frontal projection of the nasal sinuses. Maxillary sinus ostium level. (**A**) Patient 1 (norma); (**B**) Patient 2 (NSD in the left side and NTH); (**C**) Patient 3 (NSD in the right side); (**D**) Patient 4 (NSD in both side and BCB).

This study was based on the medical data of 4 patients: one healthy patient (Fig. 1A), one with nasal septal deviation (NSD) and nasal turbinate hypertrophy (NTH) (Fig. 1B), one with NSD (Fig. 1C), and one patient with bilateral concha bullosa (BCB) and NSD (Fig. 1D). The group consisted of 4 (100%) males, ranging 32–56 years of age. All of the participants underwent medical history screening. In the case of Patient 1, pre-existing nasal sinus disease, prior nasal sinus complaints, head trauma, and prior nasal surgery were excluded. In the case of Patient 2, the NSD with nasal obstruction was objectively confirmed, whereas Patient 3 had NSD without nasal obstruction. Patient 4 had NSD and bilateral concha bullosa with nasal obstruction. Pathological changes in maxillary sinuses were objectively excluded in all four patients (Fig. 1A–D). Typically, 298–445 CT slices (layers) were acquired per patient. The average CT dose index (CTDIvol) was 7.12 mGy and 7.42 mGy. The mean effective dose for all acquisitions was approximately 0.25 mSv.

**Figure 2:**
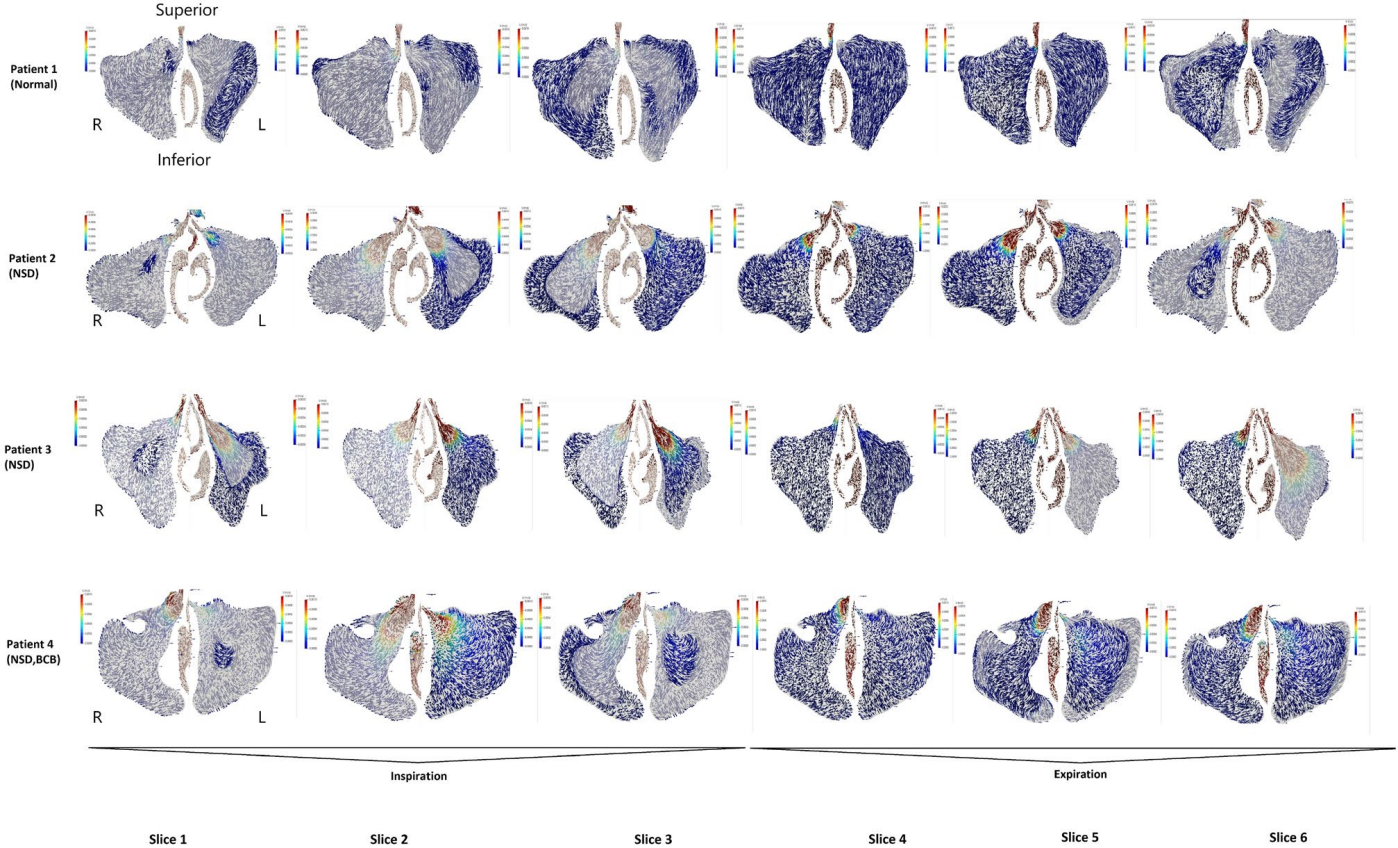
Airflow in the maxillary sinus (frontal projection, maxillary sinus ostium level). It is a visualization of the lumen of the maxillary sinuses on both sides, with the nasal cavity excised. The inspiration and expiration phases were 2 s each. For each phase, the results were presented in 3 sequences: the beginning of the phase (first 0.2–0.3 s), half point (0.9–1.0 s), the end (1.8–2.0 s).

#### Patient 1

**Figure 3:**
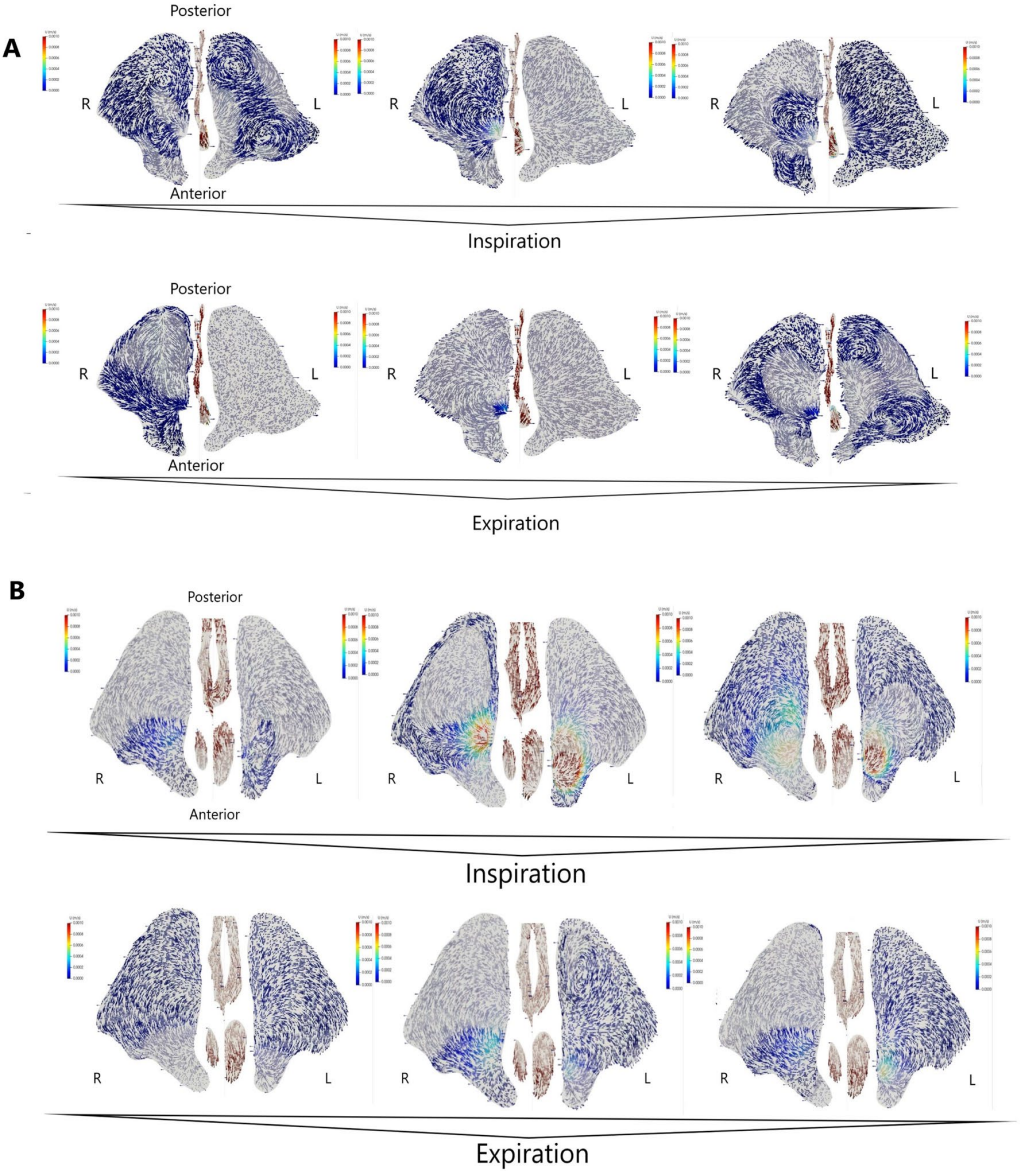
(**A**) Airflow in the maxillary sinus of Patient 1 (axial projection, middle part of maxillary sinus). Bilateral visualization of the maxillary sinus lumen, the nasal cavity excised in the middle of its height. Inspiration and expiration lasted 2 s each. Measurements were obtained at the start of each phase (first 0.2–0.3 s), half-point (0.9–1.0 s), end (1.8–2.0 s); (**B**) Airflow in the maxillary sinus of Patient 2 (axial projection, middle part of maxillary sinus). Bilateral visualization of the maxillary sinus lumen, the nasal cavity excised in the middle of its height. Inspiration and expiration lasted 2 s each. Measurements were obtained at the start of each phase (first 0.2–0.3 s), half-point (0.9–1.0 s), end (1.8–2.0 s).

No abnormalities were found during the clinical and radiological examination (Fig. 1A). The CFD analysis revealed airflow towards the sinus ostia, which reversed its direction into the sinus during the end phase of respiration. It was correlated with a reduction of airflow speed and pressure. The opposite situation during expiration was observed: initially, the air entered the sinus, and later, it reversed its direction towards the sinus ostia. Such reversal of direction occurred in the final 0.2–0.4 s (10–20%) of inspiration and expiration phases. The air flowed into the sinus along its anterior wall, later along the inferior wall, and finally the posterior wall. In contrast, the air flowing out of the sinus in the opposite sequence. Different flow speeds were noted, the highest were measured near the sinus ostia (the air exchange was the most intense at this location), and the lowest was in the peripheral parts of the sinuses. This patient’s airflow was not laminar, and numerous turbulence, complications (even pulsations), and direction changes were noted (Figs. 2, 3A). The airflow speed in the nose during inspiration and expiration varied from 1.0 to 2.5 $\text {m}\,\text {s}^{-1}$, whereas in the middle nasal meatus it was 1.0–1.5 $\text {m}\,\text {s}^{-1}$. At the ostia of the maxillary sinus (in the area of the ostiomeatal complex (OMC), the flow speed was three order of magnitude slower, about 0.001 $\text {m}\,\text {s}^{-1}$. In comparison, in the middle part of the sinus and along its walls, the flow was negligible at 0.0001 $\text {m}\,\text {s}^{-1}$. Flows in the vicinity of the sinus ostia were by an order of magnitude slower. Any significant difference in the airflows on the left and right sides of the nose and the maxillary sinus ostia was not observed (Figs. 2, 3A).

#### Patient 2

Clinical and radiological examination revealed NSD towards the left and slight bilateral CB (Fig. 1B). CFD analysis of the maxillary sinuses revealed similar flow directions and respiratory phase-related changes as in Patient 1. However, a more chaotic pattern of intrasinus flow, with numerous complications, bilateral pulsations, and direction changes even during a breathing phase, not just at the late inspiration/early expiration was noted (Figs. 2, 3B).

The flow speeds through the nose and middle nasal meatus were similar as in Patient 1. At the maxillary sinus ostia (in the OMC area), the flow speed was about 0.0008 $\text {m}\,\text {s}^{-1}$. In the same time, the flow was negligible at < 0.0001 $\text {m}\,\text {s}^{-1}$ in the middle part of the sinus and along its walls. Slightly smaller flow intensities in the area of the maxillary sinus ostia on the left side of the nose, towards which the nasal septum was deviated (Figs. 2, 3B) was noted.

#### Patient 3

**Figure 4:**
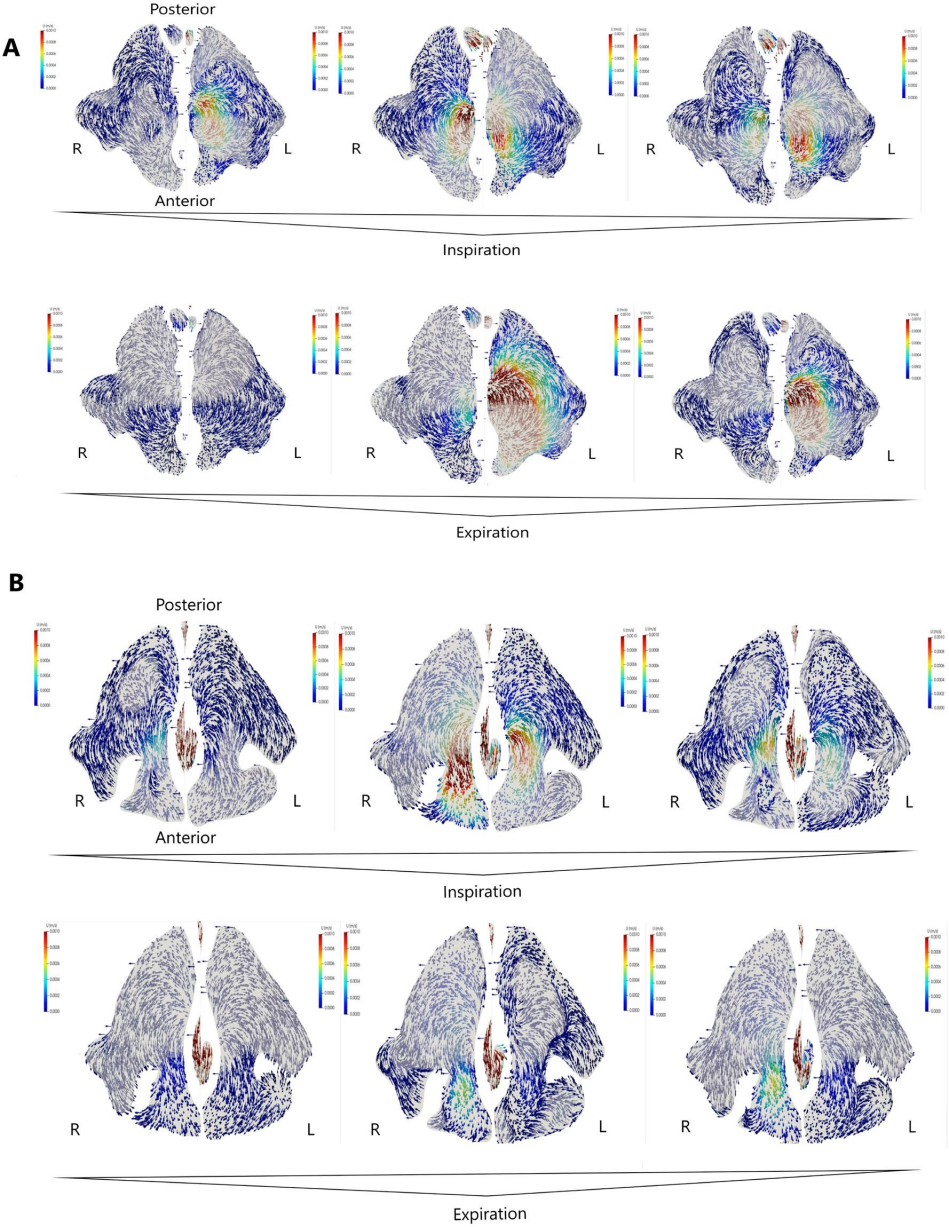
(**A**) Airflow in the maxillary sinus of Patient 3 (axial projection, middle part of maxillary sinus). Bilateral visualization of the maxillary sinus lumen, the nasal cavity excised in the middle of its height. Inspiration and expiration lasted 2 s each. Measurements were obtained at the start of each phase (first 0.2–0.3 s), half-point (0.9–1.0 s), end (1.8–2.0 s); (**B**) Airflow in the maxillary sinus of Patient 4 (axial projection, middle part of maxillary sinus). Bilateral visualization of the maxillary sinus lumen, the nasal cavity excised in the middle of its height. Inspiration and expiration lasted 2 s each. Measurements were obtained at the start of each phase (first 0.2–0.3 s), half-point (0.9–1.0 s), end (1.8–2.0 s).

Clinical and radiological examination revealed NSD towards the right (Fig. 1C). CFD results were similar to Patients 1 and 2. In addition, similar respiratory phase-dependent changes in airflow direction were observed. However, similarly to Patient 2, these flows were chaotic with bilateral pulsations and direction changes during the phases (Figs. 2, 4A). A reduced flow speed and intensity near the right side of the maxillary sinus ostia were also noted in comparison with the other side of the patient’s nasal cavity (Figs. 2, 4A).

#### Patient 4

Clinical and radiological examination revealed bilateral NSD and bilateral CB (on the right side, it was almost twice as large as on the left, 6 and 3 mm respectively) (Fig. 1D). In addition, CFD revealed numerous complications, pulsations, and direction changes similarly to patients 2 and 3. Interestingly, flow speeds and intensities near the maxillary sinus ostia were reduced on the left side despite the CB being much more prominent on the right side (Figs. 2, 4B).

The velocity vectors in Figs. 2, 3 and 4 that point toward the reader are marked with higher color saturation compared to the vectors pointing away from the reader. This allows for better visualization of the perpendicular component of velocity.

### Values of vorticity measures

**Figure 5:**
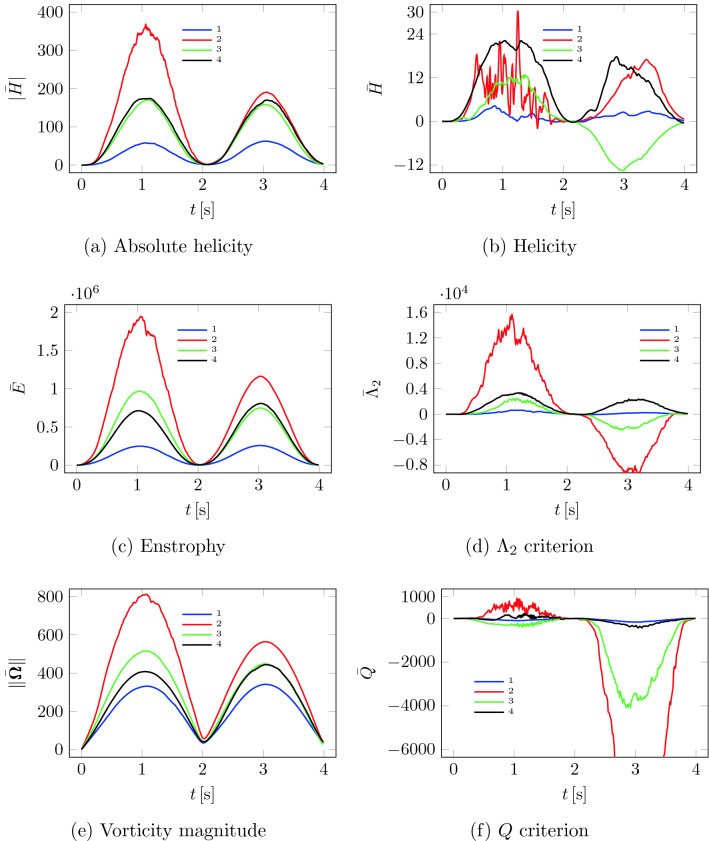
Vorticity measures as a function of time.

Individual values of vorticity measures (explained in paragraph “Concept of vorticity measures” section) are shown in Table 1. The volume integrals as a function of time are shown in Fig. 5. Vorticity measures are provided separately for inspiration (subindex ‘*i*’), exhalation (subindex ‘*e*’), and the entire breathing cycle (subindex ‘$e+i$’). For healthy patient 1, all vorticity measures took the lowest values in the sense of absolute value. This means that for patient 1 (N), the flow was the least complicated. Additionally, since the enstrophy $\bar{\bar{E}}$ values have the smallest values, the mechanical energy dissipation also has the lowest values for patient 1 (N), even though the flow volume $|V|$ was the highest in this case. In addition to the vorticity measures in Table 1, the nasal resistance values NR were given, which also have the lowest values for patient 1 (N). The worst vorticity measures (except helicity $\bar{\bar{H}}$) and nasal resistance NR were obtained for patient 2 (NSD). Vorticity measures for patients 3 (NSD) and 4 (NSD, BCB) took, in most cases, intermediate values between patient 1 (N) and 2 (NSD). Figure 5 presents individual measures’ values at a given moment in time for the entire respiratory cycle. Similarly, in this case, the individual functions are closest to zero for patient 1 (N) and furthest (except for helicity $\bar{\bar{H}}$) for patient 2 (NSD).

**Table 1:** Vorticity measures: *e*—expiration; *i*—inspiration; NR—nasal resistance; $\bar{\bar{|H|}}$—absolute helicity; $\bar{\bar{E}}$—enstrophy; $\bar{\bar{H}}$—helicity; $\bar{\bar{\Lambda }}_2$—$\lambda _2$ criterion; $\bar{\bar{Q}}$—Q-criterion; $\bar{\bar{\Vert \boldsymbol{\Omega} \Vert }}$—vorticity magnitude.

Patient	1	2	3	4
Condition	N	DSN	DSN	DSN, BCB
Sex	M	M	M	M
Age	38	33	56	32
Weight [kg]	84	89	91	97
Height [cm]	179	182	173	185
Volume $|V|$ [ml]	109.5	63.74	86.03	101.79
$\text {NR}_e \,[\text {Pa s/ml}]$	0.0133	0.0204	0.0187	0.0441
$\text {NR}_i \,[\text {Pa s/ml}]$	0.0163	0.0924	0.0635	0.0464
$\bar{\bar{|H|}}_e$	26.52	165.99	78.25	85.85
$\bar{\bar{|H|}}_i$	29.63	87.82	73.38	81.32
$\bar{\bar{|H|}}_{e+i}$	28.08	126.91	75.82	83.59
$\bar{\bar{E}}_e$	120269	892945	452650	337337
$\bar{\bar{E}}_i$	122996	543046	345020	376481
$\bar{\bar{E}}_{e+i}$	121633	717998	398836	356910
$\bar{\bar{H}}_e$	1.161	6.488	5.293	12.556
$\bar{\bar{H}}_i$	1.267	6.594	− 5.788	7.118
$\bar{\bar{H}}_{e+i}$	1.214	6.541	− 0.247	9.838
$\bar{\bar{\Lambda }}_{2e}$	242.0	6034.9	867.4	1501.9
$\bar{\bar{\Lambda }}_{2i}$	93.2	− 3692.2	− 890.0	1036.0
$\bar{\bar{\Lambda }}_{2e+i}$	167.6	1171.4	− 11.3	1268.9
$\bar{\bar{Q}}_e$	− 39.8	263.2	− 156.4	31.1
$\bar{\bar{Q}}_i$	− 73.5	− 5150.8	− 1701.9	− 159.2
$\bar{\bar{Q}}_{e+i}$	− 56.6	− 2443.8	− 929.2	− 64.1
$\bar{\bar{\Vert \boldsymbol{\Omega} \Vert }}_e$	206.5	491.8	317.6	253.4
$\bar{\bar{\Vert \boldsymbol{\Omega} \Vert }}_i$	213.2	353.4	276.5	273.2
$\bar{\bar{\Vert \boldsymbol{\Omega} \Vert }}_{e+i}$	209.9	422.6	297.0	263.3

## Discussion

The issue of airflow in the maxillary sinuses and its role in pathological processes is not conclusively described in the available literature. Chong et al. underline the role of air in transferring pathologies into the sinus cavity and the development of pathologies in case of obstruction between the nose and sinus^21^. Our study seems to be one of the first to qualitatively and quantitatively assess the airflow in the maxillary sinuses. Based on the results of our study, it is not possible to make conclusions of wide applicability. But what does one know based on them?

1. Airflow exchange into the maxillary sinus during breathing, both in the inspiratory and expiratory phases was observed. The airflow speed in the sinus ostia is over three order of magnitude lower than through the nose and was about 0.001 $\text {m}\,\text {s}^{-1}$.
2. The airflow through the sinus ostia to the sinus cavity and nasal cavity is multidirectional within one respiration phase. Both during inhalation and exhalation, it is reversed in their final stages.
3. In the case of basic pathologies of the nasal cavity, such as nasal septum deviation, concha bullosa, when they do not concern the ostiomeatal complex, the pattern of airflow in the sinus is similar to that of a healthy nose.
4. The airflow directions in the sinus ostia region and their intensity depend on nasal septum deviation and concha bullosa. The intra-sinus airflow is more chaotic in the nose with NSD and CB compared to the norm. The proposed measures of vorticity show clear correlations between the healthy nose and pathology in the nasal cavity. The values of all indices (Table 1) were the lowest for Patient 1 compared to Patients 2, 3, and 4. Apart from the noticeable results in the lowest values of NR, the minimum values were achieved for helicity $\bar{\bar{H}}$ and its modulus $\bar{\bar{|H|}}$. The same observations were in the case of the enstrophy $\bar{\bar{E}}$ and the vorticity magnitude $\bar{\bar{\Vert \boldsymbol{\Omega }\Vert }}$. It is no different in the case of $\bar{\bar{Q}}$-criterion and $\bar{\bar{\Lambda }}_2$. The comparisons made also show the correlation of the volume $|V|$ respiratory tract with individual measures. This means that the smaller the volume of the nasal cavity and the maxillary sinus, the greater the complication of the flow, which may manifest in, e.g., additional turbulence. In addition, it can lead to a more significant effect of the air on the mucosa in direct contact with the air flowing through it.
5. As in the case of mucociliary transport, one does not observe that air moves evenly throughout the sinus ostia. The term ‘secretions expressways’ also relates to the directions of the airflow. There are places where the air moves intensively (closer to the sinus of the ostia and the posterior wall of the sinus), and there are places where its movement is minimal, almost invisible^21^. The airflow in the normal maxillary sinuses is continuous. NSD and CB change the nature of the airflow to pulsating. Several changes in the airflow direction during one respiratory phase were observed. The airflow rate within the maxillary sinus ostia is lower on the side of the bulge of the nasal septum.

Another obvious limitation of our study is the small number of analyzed CT scans. The reason for this is the difficulty in preparing and importing CT images of the nose and sinuses into the computational software and the exceedingly long time (up to several days) needed to complete the flow calculations.

Our results suggest that one has much more to learn about airflow in the nasal passage and sinuses. Thus more research is needed. Future studies on large groups of patients might clarify the ranges of airflow turbulence measurements in healthy patients and those with sinus pathologies. Furthermore, such data will allow precise assessment of disease severity using dedicated software. The apparent benefits of such tools would be speed and sensitivity of the diagnostic process and more objective decision-making regarding further interventions.

## Conclusions

CFD analysis of the 3D model of the upper respiratory tract based on CT of the nasal sinuses allows for the simulation of airflow and its quantification in patients with and without respiratory tract pathology. The proposed measurements of vorticity also permit the quantitative assessment/classification of flow complications. The values of these measures make it possible to easily distinguish the geometries of the upper respiratory tract from pathological geometries both by the specialist and by automatic software. As a result of the study, it was found that the NSD in its lower part has the most significant influence on the change in airflow within the maxillary sinuses compared to concha bullosa and the NSD in its upper part. When these pathologies are present, intra-sinus flow is more chaotic than normal. In a healthy respiratory tract, the flow of air in the sinuses is continuous. The nasal septum deviation and concha bullosa make this flow pulsatile. Several changes in the direction of airflow during one respiratory phase were observed. The flow intensity within the maxillary sinus is lower on the side of the nasal septum deviation.

## Methods

### Nasal sinuses model

The craniofacial computed tomography (CT) scans were obtained from a patient reporting to the Emergency Department due to headaches (Patient 1) and patients reporting to the Otolaryngology Outpatient Clinic due to difficulties in nasal breathing (Patient 2, 3, and 4). The scans were assessed in three typical planes (sagittal, axial, and coronal). The CT images were obtained in axial planes with multiplanar reconstructions with a slice thickness of 0.6–0.75 mm, resolution of 512 $\times$ 512 pixels, and pixel size 0.3906 $\times$ 0.3906 mm. The CT scans of Patient 1 were used to create the computational model of the normal nasal cavity and nasal sinuses. Patients 2–4 had different anatomical changes in the nasal cavity causing nasal obstruction but without pathological changes in the maxillary sinuses. Image processing and model rendering was performed using 3-D Slicer and Autodesk$^{\circledR }$ Meshmixer TM. A detailed description of the model preparation process was described in our previous publication^24^. The evaluation of the flow studies was performed separately for inspiration and separately for expiration. The study focused on assessing air movement inside the maxillary sinuses and was conducted by two experienced otolaryngologists, who also interpreted the results independently.

The Regional Bioethics Committee of the Medical University of Gdansk, Poland, approved our study protocol (approval nr. NKBBN/521/2013). The research was performed in accordance with the Declaration of Helsinki. The informed consent from all participants to use their CT images in this study and publish the study results were obtained.

### Governing equations

The numerical simulation of the incompressible flow was performed using the Reynolds-Average Simulation approach. A closed system of equations consists of the mass conservation equation, the Reynolds equation^25^, and two additional transport equations for the *k*-$\omega$ SST turbulence model^26^ with an additional equation for the eddy viscosity:

1a $$\begin{aligned}&\nabla \cdot \bar{\mathbf{u}} = 0, \end{aligned}$$

1b $$\begin{aligned}&\frac{\partial \bar{\mathbf{u}}}{\partial t} + \nabla \cdot \left( \bar{\mathbf{u}} \bar{\mathbf{u}} \right) = - \nabla \left( \tfrac{p}{\rho }+ \tfrac{2}{3} k \right) + \nabla \cdot \left( \left( \nu _t + \nu \right) 2 \bar{\mathbf{D}} \right) , \end{aligned}$$

1c $$\begin{aligned}&\frac{\partial k}{\partial t} + \nabla \cdot \left( k \bar{\mathbf{u}} \right) = 2 \nu _t \bar{\mathbf{D}}^2 + \nabla \cdot \left( \left( \tfrac{\nu _t}{\sigma _{k3}} + \nu \right) \nabla k \right) - C_\mu k \omega , \end{aligned}$$

1d $$\begin{aligned}\frac{\partial \omega }{\partial t} + \nabla \cdot \left( \omega \bar{\mathbf{u}} \right) &= \alpha _3 \frac{\omega }{k} 2 \nu _t \bar{\mathbf{D}}^2 + \nabla \cdot \left( \left( \tfrac{\nu _t}{\sigma _{\omega 3}} + \nu \right) \nabla \omega \right) \nonumber \\&\quad -\beta _3 \omega ^2 + (1 - F_1) \frac{2}{\omega }\sigma _{\omega 3} \nabla k \cdot \nabla \omega , \end{aligned}$$

1e $$\begin{aligned}&\nu _t = a_1 k \, {\max }^{-1} \left( a_1 \omega , \sqrt{2 \bar{\mathbf{D}}^2} F_2 \right) , \end{aligned}$$

where $\mathbf{u}$ is the velocity vector, *p* is the pressure, $\rho$—the density, $\nu$—the kinematic viscosity coefficient, $\nu _t$—the eddy viscosity, $\mathbf{D}$—the strain-rate tensor, *k*—the kinetic energy of velocity fluctuations and $\omega$—the turbulence frequency. Furthermore, the constants marked with the subscript ‘3’, namely $\sigma _{k3}$, $\sigma _{\omega 3}$, $\alpha _3$, $\beta _3$ are linear combinations of the constants from the component models. The additional constants are $a_1 = 0.31$, $C_\mu = 0.09$. Finally, $F_1$ and $F_2$ are the two blending functions. Other possibilities such as a direct solution of the Navier–Stokes equations or transitional turbulence models are investigated for similar geometries and boundary conditions^24^.

Since all the transport equations (1) have common terms, the general transport equation for a quantity $\phi$ has the form of:

2 $$\begin{aligned} \frac{\partial \phi }{\partial t} + \nabla \cdot \left( \phi \mathbf{u} \right) = \nabla \cdot \left( \Gamma \nabla \phi \right) + S_C + S_P \, \phi \end{aligned}$$

where the overall linearized source term is $S_C + S_P \, \phi$. The diffusivity for $\phi$ is denoted as $\Gamma$. The governing equations were discretized using the finite volume method^27^ and the integral version of the transport equation (2) over a control volume $V_P$ can be expressed as:

3 $$\begin{aligned} \frac{\mathrm d \phi _P}{\mathrm d t} |V_P| + \sum _f { \phi _f \mathbf{u}_f \cdot \mathbf{S}_f} = \sum _f { \Gamma _f \left( \nabla \phi \right) _f \cdot \mathbf{S}_f} + S_C |V_P| + S_P |V_P| \phi _P. \end{aligned}$$

Divergence schemes include both convection terms $\nabla \cdot (\phi \mathbf{u})$ and other diffusive $\nabla \cdot (\Gamma \nabla \phi )$ terms and involved Gauss integration and were interpolated through cell-centered values. The discretised convection term were interpolated by means of cell centred values because the values $\phi _f$ are located at the face centroids. Second-order accurate linear upwind interpolation was used. Moreover, the discretized diffusive terms involved surface normal gradients $(\nabla \phi )_f \cdot \mathbf{S}_f$ evaluated at a cell face that connects two cells. In order to maintain second-order accuracy for non-orthogonal meshes, an additional explicit non-orthogonal and limited correction was considered. Velocity and pressure gradients took advantage of Gaussian integration and limited linear interpolation. Finally, the fluxes $\mathbf{u}_f \cdot \mathbf{S}_f$ also made use of linear interpolation. The time derivative $\frac{\mathrm d \phi _P}{\mathrm d t}$ was discretized utilizing an implicit, three-level method (backward differencing), and the transient system of equations was solved using the PISO algorithm^29^. The corrected pressure equation was solved utilizing the GAMG solver with the combined diagonal-based incomplete Cholesky and Gauss-Seidel smoother in which Gauss-Seidel follows smoothing. For quantities *k* and $\omega$ smooth solvers using a Gauss–Seidel smoother were utilized for the velocity fields and turbulence. Subsequently, the corresponding algebraic equation system was solved using the open-source software OpenFOAM^28^.

### Boundary conditions

All the walls except for the inlet and outlet were regarded as no-slip walls, i.e., zero velocity Dirichlet boundary condition was applied accompanied by a zero gradient pressure, i.e., Neumann condition. Furthermore, the flow in the region of the near walls was modeled by the scalable wall function to retain stability. As for the inlet condition localized at the larynx, the volumetric flow rate $\dot{V}$ was specified according to the following equation^24,30^:

4 $$\begin{aligned} \dot{V} = A \sin \frac{2 \pi t}{T} \quad \quad \frac{{\text {ml}}}{{\text {s}}} \end{aligned}$$

where $A=0.267 \cdot 10^3$ is the peak amplitude expressed in seconds and results in a volumetric flow rate of 5.1 liters per minute. Furthermore, *t* is the time, and *T* is the complete breathing cycle period. The typical breath took 4 seconds and was divided into 2 phases (exhalation and inhalation), both assumed to last 2 seconds. The specified volumetric flow rate also results in a uniform velocity field normal to the inlet surface. Also, the pressure gradient was set so that the velocity boundary condition specifies the flux on the boundary. Finally, low turbulence intensity was assumed to calculate turbulence quantities *k* and $\omega$.

The outlet surfaces were localized at the external nostrils where the total pressure distribution equal to atmospheric pressure was assumed, i.e., the outlet pressure was described by subtracting the dynamic pressure from the total pressure. As for the velocity boundary, a zero-gradient condition was applied for outflows, or the velocity was obtained from the patch-face normal component for inflows.

### Domain discretization

The mesh size corresponds directly to computed tomography slice thickness 0.25 mm^24^. Furthermore, the computational mesh was classified as Cartesian mesh, i.e., it consists of mostly hexahedral elements.

The four seconds of full breathing cycle period *T* was divided into 4000 fixed time steps $\Delta t$, corresponding to $\Delta t = 0.001$ seconds per step. What is more, the highest Courant number did not exceed 4. Table 2 shows the individual computing time (in hours) for CFD calculations on a Xeon 5120 2.2 GHz processor (13 out of 14 cores involved).

**Table 2:** Mesh statistics.

Patient	1	2	3	4
Nodes	9,511,076	6,519,472	7,922,434	9,625,271
Volumes	9,001,929	6,119,137	7,460,986	9,102,000
Computation [h]	13.5	9.1	11.4	13.5

**Figure 6:**
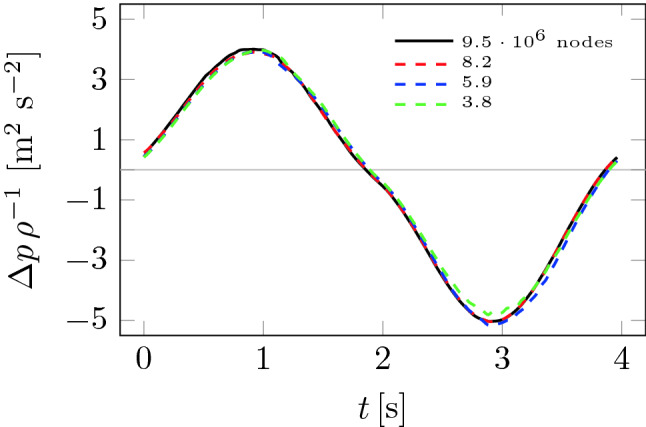
Mesh check.

A mesh study was performed for Patient 1 ranging from 3.8 to $9.5 \cdot 10^6$ nodes. Figure 6 shows the effect of the computational mesh on pressure drops during the breathing cycle. The results for the small ($3.8 \cdot 10^6$ nodes) and medium ($5.9 \cdot 10^6$ nodes) meshes are minimally different from the results for the fine mesh ($8.2 \cdot 10^6$ nodes). The pressure drops for the fine and finest ($9.5 \cdot 10^6$ nodes) meshed show virtually no differences. Therefore, it was decided to choose the finest mesh. The calculated pressure drops made it possible to determine the nasal resistance (NR) which were then compared with the data in^19^, i.e. the nasal resistances during inhalation (similar flow-rates) were in the range of 0.017–0.153 Pa s/ml, while the results in this paper take similar values: 0.016–0.092 Pa s/ml ($\text {NR}_i$ in table 1).

### Concept of vorticity measures

Vorticity measures can be used, among others, to evaluate and compare various flows or flow configurations^20^. At least two types of such measures can be distinguished. The first type relies on the vorticity vector itself $\boldsymbol{\Omega }$, i.e., the curl of velocity $\nabla \times \mathbf{u}$, whereas the second type is based on the velocity gradient $\nabla \mathbf{u}$ tensor invariants. What is important is that the former type of vorticity measure permits unambiguous determination of the fluid motion’s local topology^21^. In this study, some vorticity measures to evaluate the complication of a flow inside the upper respiratory tracts were used.

The simplest vorticity measure is the magnitude of a vorticity vector $\Vert \boldsymbol{\Omega }\Vert$:

5 $$\begin{aligned} \bar{\Vert {\boldsymbol{\Omega }}\Vert } = \frac{1}{|V|} \int _V {\Vert \boldsymbol{\Omega }\Vert \, \mathrm dV}, \end{aligned}$$

that describes the local spinning motion. Subsequently, the following measurements can be introduced through the vorticity vector $\boldsymbol{\Omega}$, such as helicity, absolute helicity or enstrophy. Typically, helicity is defined as a dot product of velocity $\mathbf{u}$ and vorticity $\boldsymbol{\Omega }$ vectors:

6 $$\begin{aligned} \bar{H} = \frac{1}{|V|} \int _V {\mathbf{u} \cdot \boldsymbol{\Omega }\, \mathrm dV} \end{aligned}$$

and is related to the topological properties of vortex lines in flow. Furthermore, absolute helicity is defined as the absolute value of helicity:

7 $$\begin{aligned} \bar{|H|} = \frac{1}{|V|} \int _V {| \mathbf{u} \cdot \boldsymbol{\Omega }| \, \mathrm dV}. \end{aligned}$$

Finally, the enstrophy is defined as one half the square of the vorticity magnitude:

8 $$\begin{aligned} \bar{E} = \frac{1}{|V|} \int _V {\tfrac{1}{2} \Vert \boldsymbol{\Omega }\Vert ^2 \, \mathrm dV} \end{aligned}$$

and is related to the kinetic energy in the flow and the dissipation power.

The most popular vorticity measure (criterion) based on the velocity gradient $\nabla \mathbf{u}$ tensor invariants is the so-called *Q*-criterion^22^:

9 $$\begin{aligned} \bar{Q} = \frac{1}{|V|} \int _V {\tfrac{1}{2} \left( \Vert \mathbf{A}\Vert ^2 - \Vert \mathbf{D}\Vert ^2 \right) \mathrm dV}. \end{aligned}$$

where $\mathbf{D}$ id symmetric and $\mathbf{A}$ antisymmetric parts of the velocity gradient tensor $\nabla \mathbf{u}$. What is more, the *Q*-criterion represents a global balance between the power of energy dissipation and enstrophy^20^. Finally, the last vorticity measure (criterion) discussed here is the $\Lambda _2$^23^. Formally, the $\Lambda _2$ is defined as the second eigenvalue $\lambda _2$ of the sum of squares of symmetric $\mathbf{D}$ and antisymmetric $\mathbf{A}$ parts of the velocity gradient tensor $\nabla \mathbf{u}$:

10 $$\begin{aligned} \bar{\Lambda }_2 = \frac{1}{|V|} \int _V {\lambda _2 \left( \mathbf{D}^2 + \mathbf{A}^2 \right) \mathrm dV}. \end{aligned}$$

The vorticity measures are understood as the integral average over time *t* of an integral average over flow volume *V*, i.e.:

11 $$\begin{aligned} \bar{\bar{f}} = \frac{1}{T} \int _0^{T} { \bar{f}(t) \, \mathrm dt} = \frac{1}{T} \int _0^{T} {\frac{1}{|V|} \int _V { f(x,y,z,t) \, \mathrm dV} \, \mathrm dt}. \end{aligned}$$

### Ethics approval

The protocol of this study was approved by the Regional Bioethics Committee at the Medical University of Gdansk, Poland (approval nr. NKBBN/521/2013).

### Consent to participate

Each patient gave written consent to use their CT images in this study.

### Consent for publication

All authors gave their final approval and agreed to be accountable for all aspects of the work.
